# *OFD1* : One gene, several disorders

**DOI:** 10.1002/ajmg.c.31962

**Published:** 2022-02-02

**Authors:** Nunziana Pezzella, Guglielmo Bove, Roberta Tammaro, Brunella Franco

**Affiliations:** ^1^ Scuola Superiore Meridionale Naples Italy; ^2^ Telethon Institute of Genetics and Medicine (TIGEM) Naples Italy; ^3^ Department of Translational Medical Sciences University of Naples Federico II Naples Italy

**Keywords:** cilia, OFD1, primary ciliary dyskinesia, variable expressivity, X inactivation, X‐linked Joubert

## Abstract

The OFD1 protein is necessary for the formation of primary cilia and left–right asymmetry establishment but additional functions have also been ascribed to this multitask protein. When mutated, this protein results in a variety of phenotypes ranging from multiorgan involvement, such as OFD type I (OFDI) and Joubert syndromes (JBS10), and Primary ciliary dyskinesia (PCD), to the engagement of single tissues such as in the case of retinitis pigmentosa (RP23). The inheritance pattern of these condition differs from X‐linked dominant male‐lethal (OFDI) to X‐linked recessive (JBS10, PCD, and RP23). Distinctive biological peculiarities of the protein, which can contribute to explain the extreme clinical variability and the genetic mechanisms underlying the different disorders are discussed. The extensive spectrum of clinical manifestations observed in *OFD1*‐mutated patients represents a paradigmatic example of the complexity of genetic diseases. The elucidation of the mechanisms underlying this complexity will expand our comprehension of inherited disorders and will improve the clinical management of patients.

## 1 THE COMPLEXITY OF CILIA AND CILIA‐ASSOCIATED DISORDERS

Cilia are organelles extending from the cell surface of almost all mammalian cells. They display a microtubule‐based structure consisting of an axoneme anchored to the cell through the basal body which originates from the centrosome. Once thought to be vestigial organelles, cilia have now been demonstrated to play a crucial role in transduction of extracellular signals and regulation of biological processes (see Nachury & Mick, 2019 for a review on the topic).

This organelle comes in two flavors: motile and immotile cilia. In immotile cilia the axoneme consist of nine pairs of microtubules originating from the basal body. In motile cilia, additional components include a central pair of microtubules and inner and outer dynein arms that fuel the movement of cilia (Reiter & Leroux, 2017). Motile cilia can be found in multiciliated cells of the respiratory epithelium, cells of the ependymal structures in the brain, and in the reproductive organs of males and females. These motile structures function by propelling cells or moving extracellular fluids or mucus. Single immotile cilia (primary cilium) can instead be found on the surface of the majority of cells where they exert sensory function contributing to the transduction of signaling pathways among which the Hedgehog signaling (Hh) is one of the most extensively cilia‐dependent pathways studied (Ho & Stearns, 2021). Emerging evidence has suggested another specific function of primary cilia in cell cycle regulation, possibly linked to the role of centrosomal centrioles in cell division and ciliogenesis. Cilia are generally assembled in G_0_/G_1_ and disassemble before mitosis in each round of the cell cycle (Kasahara & Inagaki, 2021). Both motile and immotile cilia can be detected in the embryonic node where they contribute to left–right asymmetry establishment (Hamada, 2020). In the mouse, it has been shown that cells in the central region of the node display motile cilia responsible for a clockwise rotation while sensory immotile cilia are localized in the node periphery (Hamada, 2020). The rotational movement of motile cilia determines a leftward flow of the fluid present in the embryonic node cavity that is responsible for the body asymmetry. The immotile cilia instead sense the fluid flow and trigger activation of transduction pathways (e.g., Pkd2; Hamada, 2020).

The processes underlying cilia assembly and maintenance are quite complex and the number of different genes necessary for the formation and function of ciliary structures is estimated in the order of thousands. To date, 686 transcripts have been definitively localized to cilia (Vasquez, van Dam, & Wheway, 2021) while many more are needed for ciliary functions. Dysfunction of many of these genes and proteins has been associated with inherited conditions known as ciliopathies.

Ciliopathies involving primary cilia often affect multiple organs including the central nervous system (CNS), eyes, and skeleton as well as visceral organs such as kidneys and liver (primary ciliopathies). In particular, impairment of the Hh pathway resulting from defective cilia formation is believed to contribute to skeletal and brain malformations (Loo, Pearen, & Ramm, 2021). Ciliopathies include more common conditions such as autosomal dominant polycystic kidney disease and rarer disorders such as Oral‐facial‐digital type I (OFDI: OFD type I) and Joubert (JBS) syndromes (Reiter & Leroux, 2017). Ciliary dysfunction may also result in disorders involving single organs such as retinitis pigmentosa. On the other hand, dysfunction of motile cilia results in motile ciliopathies ranging from isolated laterality defects or subfertility in males, to primary ciliary dyskinesia (PCD), a genetically heterogeneous multisystemic disorder that represents the most common motile ciliopathy with a global estimated prevalence of 1:10,000 (Wallmeier et al., 2020).

In this report, we focus on OFD1, a cilioprotein shown to be involved in the formation of cilia and establishment of left–right asymmetry, which has been implicated in primary and motile ciliopathies presenting phenotypes ranging from isolated retinitis pigmentosa to multiorgan involvement (Morleo & Franco, 2020).

## 2 THE OFD1 PROTEIN

The OFD1 protein is encoded by one of the first transcribed sequence identified on the X‐chromosome in 1983. Initially known as *CXORF5* (alias 71‐7A, DXS69E; de Conciliis et al., 1998), the transcript was subsequently renamed *OFD1* in 2001 after the identification of mutations associated with OFD type I syndrome (Ferrante et al., 2001). OFD1 is a highly pleiotropic protein with a centrosome/basal body/pericentriolar satellites localization (Giorgio et al., 2007; Romio et al., 2004; Tang et al., 2013) and in vitro and in vivo studies have demonstrated its requirement for the formation of primary cilia and for the establishment of left–right asymmetry (Ferrante et al., 2006; Singla, Romaguera‐Ros, Garcia‐Verdugo, & Reiter, 2010). Additional functions not confined to the suppression or promotion of ciliogenesis have been ascribed to the OFD1 protein (Morleo & Franco, 2020). OFD1 is involved in the control of centrioles length and distal structure (Singla et al., 2010); chromatin remodeling at DNA double strand breaks (Abramowicz et al., 2016), protein quality balance (Amato, Morleo, Giaquinto, di Bernardo, & Franco, 2014; Iaconis et al., 2017; Liu et al., 2014), and cell cycle progression (Alfieri et al., 2020). More recently, a role for OFD1 as a novel receptor in selective autophagy has also been described (Franco & Morleo, 2021; Morleo et al., 2021; Morleo & Franco, 2021). Some of the pleiotropic functions identified for this protein, such as the role in autophagy, are cilia independent. Moreover, the OFD1 protein has also been shown to display, besides the classical centrosomal/basal body/pericentriolar satellites staining, a nuclear localization (Giorgio et al., 2007) to which some of the OFD1 function can be ascribed.

## 3 OFD TYPE I SYNDROME

OFD type I (MIM #311200) is an X‐linked dominant male‐lethal condition and belongs to the genetically heterogeneous group of Oral‐facial‐digital syndromes (Franco & Thauvin‐Robinet, 2016). This condition is characterized by dysmorphic features, malformations of the oral cavity, skeletal abnormalities, and involvement of the CNS. Cystic disease more commonly affects the kidneys, but also cysts in liver, pancreas, and ovaries can be observed in affected female patients (Franco, 2008; Macca & Franco, 2009). Dysmorphic features affecting the head and face include facial asymmetry, hypertelorism, micrognathia, broadened nasal ridge, hypoplasia of the malar bone and of the nasal alar cartilage, and frontal bossing (Gorlin, Cohen, & J. r.,, & Hennekam, R. C. M., 2001). Evanescent milia are commonly visible on the face and ears and usually disappear by the third year of life (Habib, Fraitag, Couly, & de Prost, 1992). Dryness, brittleness, and/or alopecia of the scalp hair can also be observed (Reinwein, Schilli, Ritter, Brehme, & Wolf, 1966). Malformation of the oral cavity is very common and comprise clefts of the palate and median pseudoclefting of the upper lip, tongue abnormalities (hamartomas and lobulated tongue), and hyperplastic oral frenula. Thickened alveolar ridges and abnormal dentition (malposition of the maxillary canine teeth, infraocclusions, agenesis of lower lateral incisor teeth and supernumerary teeth) are additional characteristics of OFD type I (Gorlin, 1990; Gorlin et al., 2001). Skeletal, mainly digital, defects, are very frequent and include syndactyly, brachydactyly, clinodactyly, duplication of the hallux and more rarely, pre‐ or postaxial polydactyly in affected females (Gorlin, 1990; Gorlin et al., 2001; Prattichizzo et al., 2008; Thauvin‐Robinet et al., 2006). The CNS involvement is observed in ~50% of cases and consists of brain developmental anomalies (e.g., intracerebral cysts, agenesis of the corpus callosum, and cerebellar agenesis), and/or isolated cognitive defects (Bisschoff et al., 2013; Del Giudice et al., 2014). Hearing defects have also been described. Retinal involvement is rarely observed (Macca & Franco, 2009).

Conditional animal models have been generated to overcome the lethality observed in the constitutional null mutants and characterized to better dissect the mechanisms underlying this complex pathology. Based on our in vivo studies (Bimonte et al., 2011; D’Angelo et al., 2012) it is reasonable to believe that many of the clinical manifestations observed in OFD type I (e.g., the skeletal defect and the CNS involvement) are due to a defective ciliogenesis whereas others may be due to different pathogenetic mechanisms. For example, conditional inactivation of the transcript in the kidneys demonstrated that cilia are initially present in the *Ofd1*‐inactivated renal epithelial cells and disappear when renal cysts form suggesting that the disappearance of cilia could be a secondary event (Zullo et al., 2010). On the same line, we demonstrated that modulation of autophagy improves the renal cystic phenotype (by reducing the number of renal cyst and improving the renal function) in *Ofd1* mutant animals suggesting that the renal cystic disease in OFD type I is at least partially due to a non‐ciliary function of the OFD1 protein (Morleo et al., 2021).

To date, 184 different and apparently pathogenic sequence variants (point mutations 93% and macrodeletions 7%) have been detected in 234 patients in the *OFD1* gene (Table 1). The majority (186) are found in OFD type I female patients displaying the typical orofaciodigital phenotype. Most of these variants include point mutations (more commonly frameshifts in 100 patients, but also missense [24], non‐sense [26], and splicing [26]). The portion of the transcript most commonly hit by mutations leading to the OFDI phenotype include exons 3, 7, 8, 9, 13, and 16. Being an X‐linked dominant male‐lethal condition, the mutations reported above and resulting in the OFD type I phenotype in female are often lethal in males as detailed below in the Clinical spectrum of male cases with OFD1 mutations section.

**TABLE 1:** Nucleotide changes identified in the *OFD1* transcript

Exon Intron	Nucleotide change	Type of mutation	Predicted protein	No. of cases	Sex	References
Point mutations						
**In 1**	c.13‐10 T>A	Splicing		3	F	(Bisschoff et al., 2013)
**Ex 2**	c.43‐44delAG	Frameshift	p.Q16RfsX17	1	F	(Prattichizzo et al., 2008)
	c.65dupA	Frameshift	p.L23AfsX28	1	F	(Prattichizzo et al., 2008)
	c.111G>A	Splicing		1	F	(Prattichizzo et al., 2008)
	c.111G>C	Splicing		1	F	(Prattichizzo et al., 2008)
	c.63insT	Frameshift	p.K21Dfs*8	1	F	(Bisschoff et al., 2013)
	c.52G>T	Nonsense	p.E18X	1	F	(Bisschoff et al., 2013)
**In 2**	c.111+2 T>C	Splicing		2	F	(Prattichizzo et al., 2008)
	c.111+3A>G	Splicing		1	F	(Bisschoff et al., 2013)
**Ex 3**	c.115C>T	Nonsense	p.Q39X	1	F	(Del Giudice et al., 2014)
	c.121C>T	Nonsense	p.R41X	2	F	(Ferrante et al., 2001)
	c.148insG	Frameshift	p.H50Afs*26	1	F	(Bisschoff et al., 2013)
	c.149A>G	Missense	p.H50R	1	M	(Bachmann‐Gagescu et al., 2015)
	c.162_166delTGGAG	Frameshift	p.S54RfsX73	1	F	(Prattichizzo et al., 2008)
	c.221C>T	Missense	p.S74F	2	F	(Prattichizzo et al., 2008)
	c.224A>C	Missense	p.N75T	1	F	(Prattichizzo et al., 2008)
	c.235G>A	Missense	p.A79T	1	F	(Rakkolainen, Ala‐Mello, Kristo, Orpana, & Jarvela, 2002)
	c.241C>G	Missense	p.H81D	2	F	(Prattichizzo et al., 2008)
	c.243C>G	Missense	p.H81Q	2	F	(Romero et al., 2007)
	c.247C>T	Nonsense	p.Q83X	1	F	(Prattichizzo et al., 2008)
	c.225C>G	Missense	p.N75K	1	F	(Del Giudice et al., 2014)
	c.260A>G	Missense	p.Y87C	2	F^a^	(Prattichizzo et al., 2008)
	c.274 T>C	Missense	p.S92P	1	F	(Prattichizzo et al., 2008)
	c.275‐276delCT	Frameshift	p.S92Cfs*24	1	F	(Bisschoff et al., 2013)
	c.277G>T	Missense	p.V93F	2	M	(Juric‐Sekhar, Adkins, Doherty, & Hevner, 2012)
	c.290A>G	Missense	p.E97G	1	F	(Prattichizzo et al., 2008)
	c.294_312delTGGTTTGGCAAAAGAAAG	Frameshift	p.S98RfsX138	1	F	(Ferrante et al., 2001)
	c.306delA	Frameshift	p.E103KfsX42	1	F	(Faily, Perveen, Chandler, & Clayton‐Smith, 2020)
	c.312delG	Frameshift	p.V105YfsX144	1	F	(Ferrante et al., 2001)
	c.313dupG	Frameshift	p.V105GfsX116	2	F	(Prattichizzo et al., 2008)
**In 3**	c.312+delAAAGTC	Splicing		1	F	(Ferrante et al., 2001)
**Ex 4**	c.337C>T	Nonsense	p.Q113X	1	F	(Prattichizzo et al., 2008)
	c.372C>G	Nonsense	p.Y124X	1	F	(Prattichizzo et al., 2008)
	c.358A>G	Missense	p.T120A	1	M	(Chen et al., 2018)
**In 4**	c.382‐3C>G	Splicing		1	F	(Prattichizzo et al., 2008)
	c.382‐2A>G	Splicing		1	F	(Prattichizzo et al., 2008)
**Ex 5**	c.400_403delGAAA	Frameshift	p.E134IfsX143	4	F^a^	(Del Giudice et al., 2014; Prattichizzo et al., 2008)
	c.411delA	Frameshift	p.G138VfsX144	1	F	(Prattichizzo et al., 2008)
**In 5**	c.412+2delT	Splicing		1	F	(Prattichizzo et al., 2008)
	c.412G>A	Missense	p.G138S	1	F	(Thauvin‐Robinet et al., 2006)
	c.413‐10 T>G	Splicing		1	F	(Rakkolainen et al., 2002)
**Ex 6**	c.431dupT	Frameshift	p.L144FfsX154	1	F	(Prattichizzo et al., 2008)
	c.431 T>A	Nonsense	p.L144X	1	F	(Thauvin‐Robinet et al., 2006)
	c.454C>T	Nonsense	p.Q152X	1	F	(Prattichizzo et al., 2008)
	c.422 T>G	Missense	p.M141R	2	F	(Bisschoff et al., 2013)
	c.505_506delAG	Frameshift	p.D170fs	1	F	(Fujimaru et al., 2018)
	c.506_507delGA	Frameshift	p.N170Efs*4	1	F	(Del Giudice et al., 2014)
	c.508‐509delGA	Nonsense	p.D170X	1	F	(Chetty‐John et al., 2010)
	c.515 T>C	Missense	p.L172P	1	M^a^	(Aljeaid, Lombardo, Witte, & Hopkin, 2019)
**In 6**	c.518‐1G>A	Splicing		1	F	(Bisschoff et al., 2013)
**Ex 7**	c.537_539del	Frameshift	p.D181del	1	M	(Suzuki et al., 2016)
	c.539A>T	Frameshift	p.D180Val	1	M	(Sakakibara et al., 2019)
	c.541dupG	Frameshift	p.D181Gfs*22	1	F	(Bisschoff et al., 2013)
	c.594_598delAAAGC	Nonsense	p.L200X	1	F	(Prattichizzo et al., 2008)
	c.599 T>C	Missense	p.L200P	1	M	(Y. Zhang et al., 2021)
	c.602delA	Frameshift	p.N201MfsX207	1	F	(Prattichizzo et al., 2008)
	c.604_609delGAGTAT	Frameshift	p.E202_Y203del	1	M	(Westerfield et al., 2015)
	c.607‐610delTATA	Frameshift	p.Y203Rfs*4	1	F	(Bisschoff et al., 2013)
	c.614‐617delGAGA	Frameshift	p.R205Yfs*18	1	F	(Bisschoff et al., 2013)
	c.615_620delAGAAAT	Inframe del	p.E206I207del	1	F	(Thauvin‐Robinet et al., 2006)
	c.616_617delGA	Frameshift	p.E206NfsX222	1	F	(Prattichizzo et al., 2008)
	c.628C>T	Nonsense	p.Q210X	1	F	(Prattichizzo et al., 2008)
	c.635G>C	Missense	p.R212P	2	F	(Faily et al., 2020)
	c.653delA	Frameshift	p.K218SfsX219	1	F	(Prattichizzo et al., 2008)
**In 7**	c.654+2_654+4delTA	Splicing		1	F	(Prattichizzo et al., 2008)
**Ex 8**	c.675delC	Frameshift	p.E226RfsX227	1	F	(Thauvin‐Robinet et al., 2006)
	c.702insA	Frameshift	p.Y238VfsX239	1	F	(Romio et al., 2003)
	c.707_719delAAAAGTATGAAAA	Frameshift	p.K236RfsX238	1	F	(Romio et al., 2003)
	c.709_710delAA	Frameshift	p.K237VfsX238	1	F	(Prattichizzo et al., 2008)
	c.710delA	Frameshift	p.K237SfsX242	3	F	(Alby et al., 2018; Prattichizzo et al., 2008)
	c.710dupA	Frameshift	p.Y238VfsX239	11	F	(Del Giudice et al., 2014; Prattichizzo et al., 2008)
	c.712delT	Frameshift	p.Y238MfsX242	1	F	(Thauvin‐Robinet et al., 2006)
	c.790dupG	Frameshift	p.E264GfsX269	1	F	(Prattichizzo et al., 2008)
	c.823C>T	Nonsense	p.Q275X	1	F	(Prattichizzo et al., 2008)
	18‐bp deletion	Inframe del	p.230‐235del IKMEAK	2	M	(Field et al., 2012)
**Ex 9**	c.837_838delAA	Frameshift	p.K280RfsX307	2	F	(Prattichizzo et al., 2008)
	c.967delA	Frameshift	p.S323Afs*2	1	M^b^	(Schoch et al., 2020)
	c.837_841delAAAAG	Frameshift	p.K280NfsX306	1	F	(Prattichizzo et al., 2008)
	c.839_840delAA	Frameshift	p.K280RfsX307	1	F	(Prattichizzo et al., 2008)
	c.840_844delAGAAA	Frameshift	p.K280NfsX27	2	F	(Iijima et al., 2016)
	c.843_844delAA	Frameshift	p.E281DfsX307	1	F	(Romio et al., 2003)
	c.858delG	Frameshift	p.R286SfsX290	1	F	(Prattichizzo et al., 2008)
	c.877_878delAT	Frameshift	p.M293GfsX307	4	F	(Prattichizzo et al., 2008)
	c.895insGA	Frameshift	p.A310KfsX304	1	F	(Thauvin‐Robinet et al., 2006)
	c.895‐896insGA	Frameshift	p.A301Kfs*4	1	F	(Halleux et al., 2011)
	c.914‐915delAA	Frameshift	p.Q305Sfs*2	1	F	(Del Giudice et al., 2014)
	c.871A>T	Nonsense	p.K291X	1	F	(Prattichizzo et al., 2008)
	c.919delG	Frameshift	p.V307LfsX312	1	F	(Thauvin‐Robinet et al., 2006)
	c.920 T>A	Missense	p.V307D	1	M	(Srour et al., 2015)
	c.929 T>C	Missense	p. F310S	1	M^a^	(Alamillo et al., 2015)
	c.950A>G	Frameshift	p.Q317R	1	F	(Brauner, Bignon‐Topalovic, Bashamboo, & McElreavey, 2021)
	c.951G>T	Frameshift	p.Q317H	1	F	(Brauner et al., 2021)
**In 9**	IVS9+706A>G	Cryptic ex Frameshift	p.N313fsX330	1	M	(Webb et al., 2012)
**Ex 10a**	c.1056C>G	Missense	p.N352K	1	F	(Prattichizzo et al., 2008)
**In 10**	c.1051‐2A>G	Splicing		1	F	(Prattichizzo et al., 2008)
	c.1056‐2A>T	Splicing		1	F	(Romio et al., 2003)
**Ex 11**	c.1059 T>A	Nonsense	p.Y353X	1	F	(Del Giudice et al., 2014)
	c.1071‐1078del GAAGGATG/insTTTTTCCT	Missense	p.KDD357_359del/FSY 357_359ins	1	F	(Ferrante et al., 2001)
	c.1081 T>C	Missense	p.Y361H	1	M^b^	(Pavanello et al., 2021)
	c.1099C>T	Nonsense	p.R367X	3	F	(Del Giudice et al., 2014; Prattichizzo et al., 2008, Halleux et al., 2011)
	c.1100G>A	Missense	p.R367Q	1	F	(Prattichizzo et al., 2008)
	c.1103‐1106delTGAT	Frameshift	p.L368fsX18	1	F	(Halleux et al., 2011)
	c.1128A>G	Splicing		1	F	(Del Giudice et al., 2014)
	c.1129delG	Inframe del	p.E377del	1	F	(Chetty‐John et al., 2010)
	c.1129+4A>T	Frameshift	p.T353Kfs*13/p.K354Nfs*4	1	M	(Wentzensen et al., 2016)
**In 11**	c.1130‐20_1,130‐17delAATT	Splicing		2	F	(Bisschoff et al., 2013) (Prattichizzo et al., 2008)
	c.1130‐1G>A	Splicing		1	F	(Bisschoff et al., 2013)
**Ex 12**	c.1178dupA	Frameshift	p.E394GfsX407	1	F	(Prattichizzo et al., 2008)
	c.1178del	Frameshift	p.K393Rfsx8	1	F	(Alby et al., 2018)
	c.1190dupA	Frameshift	p.N397Kfs11	1	F	(Bisschoff et al., 2013)
	c.1185delA	Frameshift	p.E395DfsX400	1	F	(Prattichizzo et al., 2008)
	c.1193_1196delAATC	Frameshift	p.Q398LfsX400	4	F	(Del Giudice et al., 2014; Prattichizzo et al., 2008)
	c.1220_1221+1delAGG	Frameshift	p.E407AfsX408	1	F	(Prattichizzo et al., 2008)
**In 12**	1221+1delG	Splicing		1	F	(Prattichizzo et al., 2008)
**Ex 13**	c.1268_1272delAAAAC	Frameshift	p.Q423PfsX428	2	F	(Prattichizzo et al., 2008)
	c.1303A>C	Missense	p.S434R	1	F	(Ferrante et al., 2001)
	c.1318delC	Nonsense	p.L440X	1	F	(Prattichizzo et al., 2008)
	c.1319delT	Frameshift	p.L440QfsX469	1	F	(Prattichizzo et al., 2008)
	c.1322_1326delAAGAA	Frameshift	p.K441RfsX450	1	F	(Prattichizzo et al., 2008)
	c.1323_1326delAGAA	Frameshift	p.E442RfsX468	3	F	(Iijima et al., 2019; Prattichizzo et al., 2008)
	c.1334_1335delTG	Frameshift	p.L445RfsX451	1	F	(Prattichizzo et al., 2008)
	c.1348‐1349delCA	Frameshift	p.Q450KfsX2	1	F	(Rotunno et al., 2019)
	c.1358 T>A	Nonsense	p.L453X	1	F	(Prattichizzo et al., 2008)
	c.1360_1363delCTTA	Frameshift	p.L454NfsX468	1	F	(Thauvin‐Robinet et al., 2006)
	c.1363‐1366del	Frameshift	p.K45SNfs*13	1	F	(Bisschoff et al., 2013)
	c.1409delA	Frameshift	p.N470TfsX472	1	F	(Rakkolainen et al., 2002)
**Ex 14**	c.1420C>T	Nonsense	p.Q474X	1	F	(Prattichizzo et al., 2008)
	c.1445_1446delTT	Frameshift	p.F482SfsX495	1	F	(Prattichizzo et al., 2008)
	c.1452_1458delAGAACTA	Frameshift	p.K484NfsX491	1	F	(Prattichizzo et al., 2008)
	c.1468G>T	Nonsense	p.E490X	1	F	(Bisschoff et al., 2013)
**Ex 15**	c.1587delA	Frameshift	p.A530LfsX532	1	F	(Thauvin‐Robinet et al., 2006)
	c.1612C>T	Nonsense	p.Q538X	1	F	(Bisschoff et al., 2013)
**Ex 16**	c.1757delG	Frameshift	p.S586MfsX590	1	F	(Ferrante et al., 2001)
	c.1821delG	Frameshift	p.I608SfsX628	1	F	(Thauvin‐Robinet et al., 2006)
	c.1840delG	Frameshift	p.A614Hfs*15	1	F	(Bruel et al., 2017)
	c.1859_1860delC	Frameshift	p.S620Cfs*8	1	F	(Bisschoff et al., 2013)
	c.1990dupC	Frameshift	p.L665Tfs*35	1	F	(Bisschoff et al., 2013)
	c.1887_1888insAT	Frameshift	p.N630IfsX666	1	F	(Rakkolainen et al., 2002)
	c.1964‐1965delG	Nonsense	p. R654X	3	F	(Dehghan Tezerjani et al., 2016)
	c.1979_1980delCT	Frameshift	p.S660CfsX	3	F	(Prattichizzo et al., 2008)
	c.2044dupA	Frameshift	p.I682NfsX700	1	F	(Prattichizzo et al., 2008)
	c.2056delT	Frameshift	p.S686PfsX717	1	F	(Prattichizzo et al., 2008)
	c.2101C>T	Nonsense	p.Q701X	1	M^a^	(Bouman et al., 2017)
	c.2122‐2125dupAAGA	Nonsense	p.N711KfsX713	2	M	(Budny et al., 2006)
	c.2176delC	Frameshift	p.R726AfsX516	1	F	(Prattichizzo et al., 2008)
	c.2183delG	Frameshift	p.G728Afs*89	1	F	(Diz et al., 2011)
**In 16**	c.2260+2 T>G	Splicing		2	M	(Sakakibara et al., 2019)
	c.2261‐1G>T	Splicing		1	F	(Prattichizzo et al., 2008)
**Ex 17**	c.2321‐2322insT	Frameshift	p.S790P*X802	1	M	(Linpeng et al., 2018)
	c.2349delC	Frameshift	p.I784SfsX816	1	F	(Thauvin‐Robinet et al., 2006)
**In 17**	c.2388+2 T>C	Splicing		2	M, F	(Tsurusaki et al., 2013)
**In 18**	c.2488+27>C	Splicing		1	M	(Linpeng et al., 2018)
**Ex 19**	c.2524G>A	Missense	p.G842R	2	M	(H.‐W. Zhang, Su, & Yao, 2020)
	c.2582dupT	Splicing		1	M	(Linpeng et al., 2018)
**Ex 20**	c.2600‐18_2600 delinsACCT	Frameshift	p.S867D869delinsN	1	M	(Sakakibara et al., 2019)
	c.2615–2619 delAAATT	Frameshift	p.Q872fs*26	1	M	(Bukowy‐Bieryllo et al., 2019)
	c.2629‐2632del	Frameshift	p.E878Kfs*9	1	M	(Suzuki et al., 2016)
	c.2632‐2635delGAAG	Inframe del	p.E878del	1	M	(Linpeng et al., 2018)
	c.2656delC	Frameshift	p.G886Kfs*2	1	M	(Kane et al., 2017)
	c.2746‐2747insT	Frameshift	p.Y916fs*7	1	M	(Bukowy‐Bieryllo et al., 2019)
	c.2767delG	Frameshift	p.E923Kfs	1	M	(Coene et al., 2009)
**Ex 21**	c.2789‐2793del TAAAAA	Frameshift	p.I930Kfs*8	1JBS10;1PCD	M^c^	(Hannah et al., 2019; Thauvin‐Robinet et al., 2013)
	c.2797dupG	Frameshift	p.E933Gfs*7	1	M	(Thauvin‐Robinet et al., 2013)
	c.2797G>T	Nonsense	p.E933X	1	M	(Bukowy‐Bieryllo et al., 2019)
	c.2815G>T	Nonsense	p.E939X	1	M	(Bukowy‐Bieryllo et al., 2019)
	c.2841_2847del	Frameshift	p.K948NfsX8	1	M	(Coene et al., 2009)
	c.2843‐2844delAA	Frameshift	p.K948RfsX	1	M	(Meng et al., 2017)
	c.2844‐2850del	Frameshift	p.K948Nfs*9	1	M	(Coene et al., 2009)
	c. 2862dupT	Nonsense	p.Q995X	1	M	(Hannah et al., 2019)
	c.2868delT	Frameshift	p.P957Lfs*2	1	M	(Hannah et al., 2019)
**Ex 22**	c.2953G>A	Missense	p.G985S	1	M	(Wang, Zheng, Liu, & Yang, 2017)
Macrodeletions						
**Ex 5**	c.381‐?_412 +?del	Deletion			F	(Thauvin‐Robinet et al., 2009)
**Ex 7–9** **In 9**	c518‐?_935 +?del	Deletion			F	(Morisawa et al., 2004)
**Ex 7–10**	c.518‐?_936‐?del	Deletion			F	(Del Giudice et al., 2014)
**Ex 11**	c.1056‐?del	Deletion			F	(Del Giudice et al., 2014)
**Ex 10–11**	c.936‐?_1129 +?del	Deletion			F	(Thauvin‐Robinet et al., 2009)
**Ex 17**	c.2261‐?_2387 +?del	Deletion			F	(Thauvin‐Robinet et al., 2009)
**Ex 16, 17, 19**	c.1654+8332599+423del	Deletion			M	(Sharma, Kalish, Goldberg, Reynoso, & Pradhan, 2016)
**Ex 13–23**	c.1222‐?_3038 +?del	Deletion			F	(Thauvin‐Robinet et al., 2009)
**Ex 1–8**	c.?_‐311_828 +?del	Deletion			F	(Thauvin‐Robinet et al., 2009)
**Ex 1–14**	c.?_‐311_1542 +?del	Deletion			F	(Thauvin‐Robinet et al., 2009)
**Entire gene**	c.1‐3039del	Deletion			F	(Bisschoff et al., 2013)
**Entire gene**	c.1‐3039del	Deletion			F	(Bisschoff et al., 2013)
**Entire gene**	c.1‐3039del	Deletion			M^a^	(Kehrer et al., 2016)

Abbreviations: Ex, exon; In, Intron.

^a^ Male and female fetuses aborted or for which the pregnancy was terminated.

^b^ Nucleotide changes not convincingly responsible for the phenotype.

^c^ The same mutation was identified associated to a PCD (Hannah et al., 2019) and JBTS10 (Thauvin‐Robinet et al., 2013) phenotype. Mutations resulting in OFD type I are indicated in light orange; JBS10 in light blue; RP23 in red; PCD in light green; in dark green is a mutation associated to clinical signs of PCD and OFD type I; in gray is a mutation associated to both PCD and JBS10.

A schematic representation of the localization of all the mutations so far identified in OFD type I patients is provided as Supporting Information Material S1. Larger genomic rearrangements involving single or multiple exons, or the entire transcript have been also described (Table 1).

A clear genotype–phenotype correlation has not yet been established and female patients display extensive intrafamilial and interfamilial clinical variability. Examples of familial cases in which the mother and the daughter displaying the same mutation show a phenotype of different severity have been reported. This variable clinical expressivity may also be influenced by the pattern of X‐inactivation as skewed X‐inactivation was observed in 30% of familial cases (Morleo & Franco, 2008; Thauvin‐Robinet et al., 2006).

## 4 CLINICAL PHENOTYPES ASSOCIATED WITH MUTATIONS IN *OFD1*

Over the years, mutations in the *OFD1* transcript were also shown to contribute to other genetic conditions associated with cilia dysfunction.

In 2006, a hemizygous mutation was reported in a family with a novel X‐linked recessive syndrome comprising intellectual disabilities, macrocephaly, obesity, skeletal abnormalities, and ciliary dysfunction. This condition was classified as Simpson Golabi Behmel syndrome type 2 (SGBS2; Budny et al., 2006). However, subsequent reports have provided experimental evidence indicating that the locus for SGBS2 in Xp22 is associated with mutations in *PIGA* and that mutations in *OFD1* should not be considered a cause of SGBS2 (Fauth & Toutain, 2017). In the original family described by Budny et al. (2006), however, all nine affected males except the index case died from respiratory problems in infancy. The respiratory problems prompted the study of cilia motility in epithelial cells from the respiratory tract and high‐speed video analysis revealed a dyskinetic beating pattern responsible for the primary ciliary dyskinesia phenotype in this family. This finding represented the first indication of a role for the OFD1 protein also in motile cilia function (Budny et al., 2006).

In 2009, the *OFD1* transcript was also implicated in an X‐linked recessive form of Joubert syndrome (JBS10; MIM #300804), a ciliopathy affecting primary cilia (Coene et al., 2009). To date a total of 23 patients have been described for this specific form (12 frameshifts, six missense, three inframe deletion, and two splicing mutations; Table 1). JBS is a recessively inherited neurodevelopmental disorder and is characterized by the presence of a specific cerebellar and brainstem malformation known as the “molar tooth sign.” Besides the neurological involvement, JBS is associated with clinical features resulting from progressive involvement of the retina, kidneys, and liver (Parisi, 2019). In JBS10, hemizygous male patients are affected, and heterozygous carrier females are asymptomatic. Many of the JBS10 male individuals reported were severely malformed and died during pregnancy or the parents decided to terminate the pregnancy. A few patients however reached early childhood and displayed polydactyly, renal cystic disease, and abnormalities in left–right determination besides brain malformations and cognitive impairment.

Later on, in 2012, a deep intronic mutation was also demonstrated to segregate in a five generation family with a severe form of X‐linked retinitis pigmentosa (RP23; MIM #300424; Webb et al., 2012; Table 1). No extraocular clinical manifestations were reported in this large family. A second report confirmed the pathogenicity of the findings describing a p.G985S variant in a 6‐year‐old boy with retinitis pigmentosa and bilateral idiopathic demyelinating optic neuritis. In this patient, highly progressive binocular vision loss occurred within 38 days and again no extraocular findings were described (Wang et al., 2017). A third missense mutation (c.358A>G; p.T120A) was reported in a patient in which the only clinical finding consisted of early onset nyctalopia and rapidly progressing retinitis pigmentosa with macular degeneration (Chen et al., 2018; Table 1).

**FIGURE 1:**
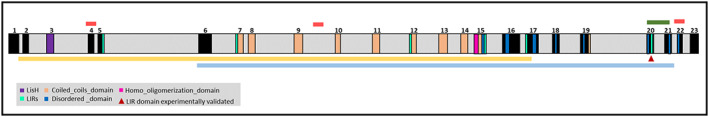
A schematic representation of the OFD1 gene is shown. Exons and introns are in scale and indicated in black and gray, respectively. The exon’s number is indicated above exons. Colored bars represent the localization of mutations per each disease phenotypes. Yellow bar OFD type I; light blue bar, JBS10; red bars, RP23; green bar, PCD patients. Protein domains are indicated in the coding region and detailed in the scheme. The LIR domain indicated with a red triangle has been experimentally validated (Morleo et al., 2021)

More recently, mutations in the *OFD1* transcript were also identified in patients affected by PCD (MIM #244400), which is, as mentioned before, a motile ciliopathy mainly affecting the respiratory epithelium and the reproductive tract. Seven different mutations have been identified to date, all affecting the C‐terminal part of the protein (Table 1; Figure 1). These patients displayed the typical early onset recurrent obstructive bronchitis, bronchiectasis, sinusitis, and otitis media. In the majority of patients, motile respiratory cilia were shown to have a normal structure and in two individuals high‐speed video microscopy demonstrated sparse cilia with stiff beating and reduced bending (Bukowy‐Bieryllo et al., 2019; Hannah et al., 2019).

Concerning the extra respiratory clinical signs, in four patients a mild intellectual disability was described, two patients presented polydactyly and two *situs inversus*. These observations suggest a contribution of dysfunction of both motile and immotile cilia to the phenotype observed.

Although all the conditions can be classified as ciliopathies, OFD type I and JBS10 involve the dysfunction of primary cilia, RP23 is a retinitis pigmentosa presumably involving dysfunction of the photoreceptor connecting cilium and PCD is a motile ciliopathy. Table 2 summarizes the clinical similarities and differences observed in these conditions.

**TABLE 2:** Clinical similarities and differences among diseases caused by *OFD1* mutations

Disease OMIM #	OFDI 311200	JBS10 300804	PCD 244400	RP23 300424
Inheritance	XLD	XLR	XLR	XLR
Sex	F	M	M	M
Craniofacial Abnormalities	+	+	−	−
Skin, nails, and hair Defects	+	−	−	−
Oral abnormalities	+	+	−	−
Retinal dysfunction	−	+	−	+
Cystic disease	+	+	−	−
Respiratory infections	−	−	+	−
Skeletal defects	+	+	+	−
CNS malformations	+	+	−	−
MTS	−	+	−	−
Cognitive impairment	+	+	+	−
*Situs Inversus*	−	+	+	−

Abbreviations: F, Female; M, Male; XLD, X‐linked dominant; XLR, X‐linked recessive.

## 5 CLINICAL SPECTRUM OF MALE CASES WITH OFD1 MUTATIONS

**FIGURE 2:**
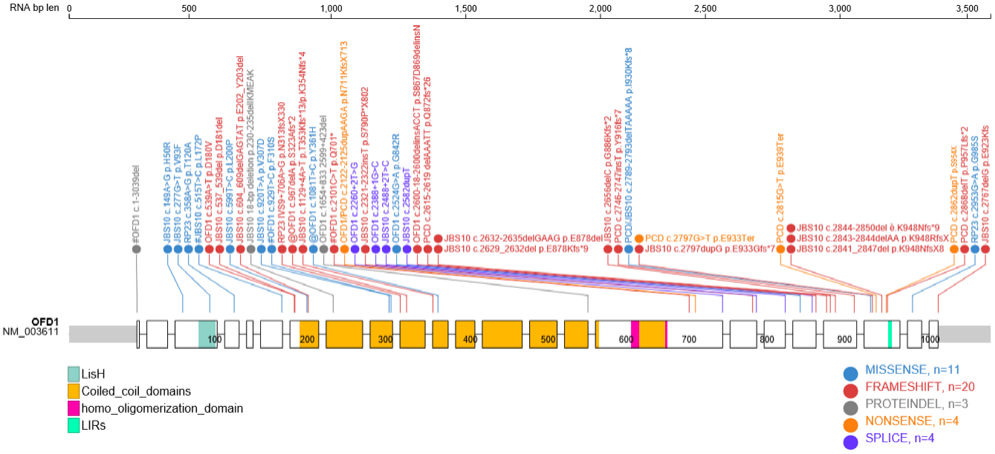
Schematic representation of the localization of the nucleotide changes identified in the OFD1 gene in male individuals. The program Protein paint (https://proteinpaint.stjude.org/) was used. Top, RNA length and base pairs are indicated. Bottom, frameshifts, missense, non‐sense, splicing, and indel mutations are reported according to the color code. The domains are shown and indicated following the nomenclature reported. Only the experimentally validated LIR in exon 21 is reported. Exons are represented as rectangles of different size, number within exons indicate positions of aminoacids. Exons, domains, RNA and mutations are in scale. # fetus, @ nucleotide changes nonconvincingly responsible for the phenotype. OFDI, OFD type I; JBS10, X‐linked recessive Joubert syndrome; RP23, retinitis pigmentosa; PCD primary ciliary dyskinesia

To date, no male individuals with the full‐blown phenotype characteristic of OFD type I syndrome and displaying mutations in the *OFD1* transcript have been described. Mutated males present either with JBS10, RP23, or PCD as described above and illustrated in Table 1 and Figure 2. There are however few cases in which male individuals with *OFD1* mutations survive and present a phenotype characterized only by some of the clinical features of OFD type I, JBS10, RP23, or PCD or with a clinical phenotype which is a combination of the different diseases (Figure 2). Budny et al. (2006) were the first to describe a recessive mutation in exon 16 (c.2122‐2125dupAAGA; p.N711KfsX713) in hemizygous males from a multigeneration Polish family displaying developmental delay, dysmorphic features, recurrent respiratory infections, digital anomalies. Functional studies demonstrated a motile cilia defect (Budny et al., 2006). Sakakibara et al. (2019) described *OFD1*‐mutated male individuals including two individuals from the same family with a splicing variant (c.2260+2T>G) in intron 16. One of the two patients displayed autistic behavior, short stature and renal failure while the other displayed obesity, intellectual disability, micropenis, and end stage renal failure. Unfortunately, the etiopathogenesis of the renal disease affecting also other members of the family was not specified. The same paper reported on an 8‐year old boy with a deletion/insertion mutation in exon 20 (c.2600‐18_2600delinsACCT; p.S867_D869delinsN) only displaying renal cystic disease and a 4‐year old boy with a missense variant in exon 7 (c.539A>T; p.D180V) showing macrocephaly, brachydactyly, brain malformation, and intellectual disabilities (Sakakibara et al., 2019). Another example is represented by a couple of 14‐year old male twins with a missense mutation (c.2524G>A; p.G842R) and renal cystic disease as the only clinical sign described by H.‐W. Zhang et al. (2020). Lastly, Sharma et al. (2016) reported the peculiar case of a 9‐year old boy with a 7.9 deletion encompassing exons 16, 17, and 19 of the OFD1 transcript associated with a complex phenotype characterized by short stature, hearing loss, small kidneys resulting in renal failure (no cysts), retinal dystrophy, and intellectual disabilities in the presence of a normal brain MRI.

## 6 OPEN QUESTIONS

Experimental evidence gathered in the last few years has demonstrated several examples of pleiotropic proteins involved in different phenotypes. So far mutations in the transcript encoding for this protein have been associated with distinct disorders that in some cases present overlapping clinical signs involving multiple organs whereas in other individuals, only specific tissues are affected. The OFD1 protein, however, displays several peculiarities.

### 6.1 X‐chromosome inactivation in OFD type I syndrome

**FIGURE 3:**
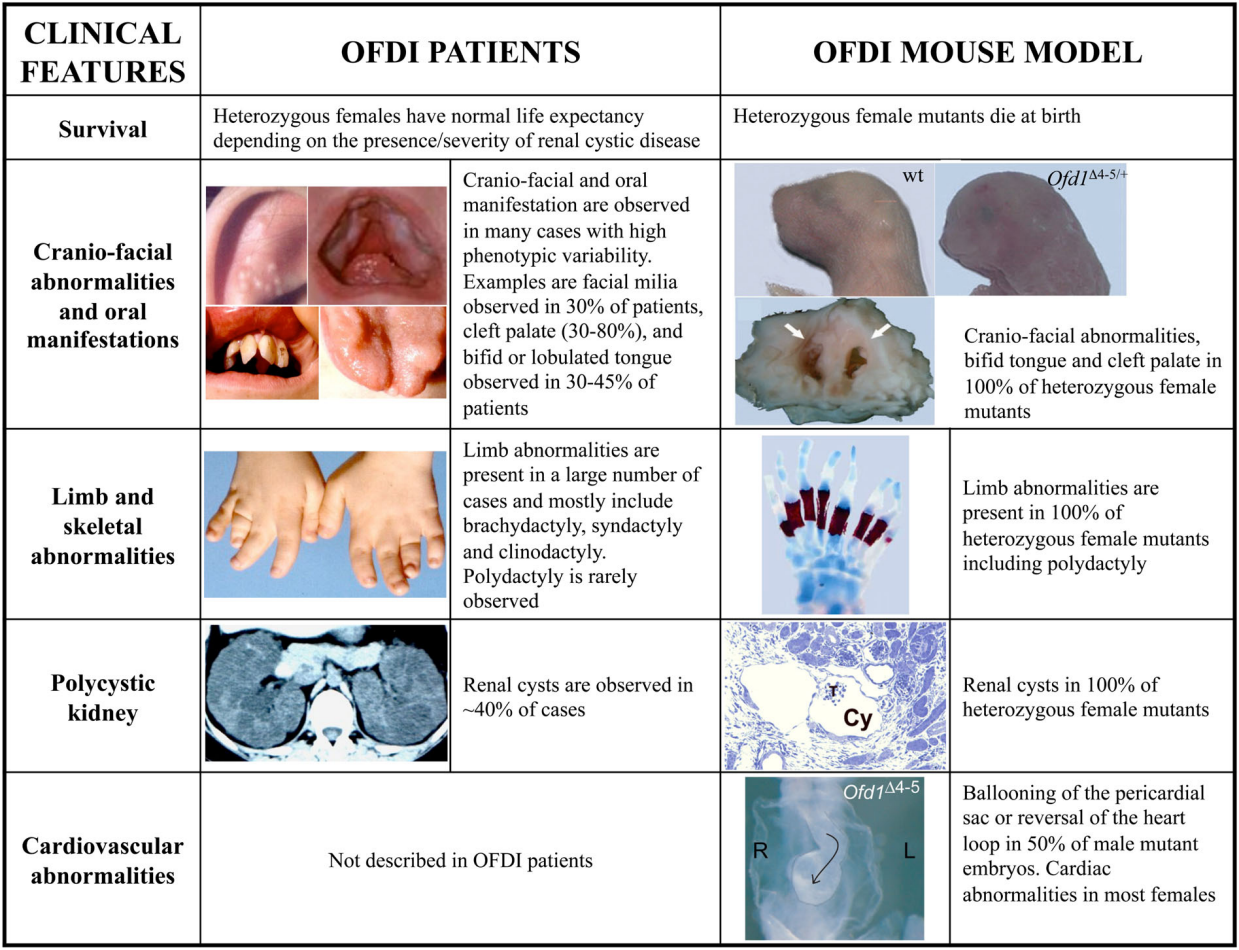
Comparison between the clinical manifestations observed in OFD type I patients and in the murine model for OFD type I syndrome. The figure illustrates that the phenotype observed in mice is more severe than that observed in humans: newborn *Ofd1* ^Δ4–5/+^ females die at birth while female patients have normal life expectancy depending on the presence/severity of the cystic disease and the CNS involvement. Concerning the cranio‐facial‐oral abnormalities, these are present in 100% of heterozygous mice analyzed (palatoschisis is marked by white arrows), while patients display an evident phenotypic variability. Examples of this are signs such as facial milia (ear), bifid lobulated tongue, teeth abnormalities and cleft palate. Limb and skeletal abnormalities are also very variable among patients while polydactyly is always present in female mutants as revealed by alizarin red (bone) and alcian blue (cartilage) staining. Cystic kidney is present in about 40% of patients while renal cysts were observed in all mutant animals. Finally cardiovascular abnormalities were observed in most mutant animals analyzed to date, both females and male embryos, while these anomalies have been rarely reported in OFD type I patients (from Morleo & Franco, 2008, with permission)

The *OFD1* gene is localized on the X chromosome and as such is present in females in two copies, one of which should undergo silencing to achieve gene dosage balance between males and females. This transcript however is part of the group of X‐chromosome genes that escape X‐chromosome inactivation (XCI) in humans (de Conciliis et al., 1998), whereas the murine counterpart is subject to the silencing of one of the two X chromosomes in females (Ferrante et al., 2003). A mouse model in which the *Ofd1* transcript had been constitutively inactivated was generated. Characterization of this model demonstrated that the complete absence of *Ofd1* in males results in early lethality with developmental defects affecting the neural tube, the heart and the correct establishment of left–right asymmetry, the latter due to defective cilia at the embryonic node. Heterozygous females die at birth and show craniofacial abnormalities and cleft palate in addition to highly penetrant renal cystic disease and skeletal abnormalities (Ferrante et al., 2006). The effects of the disruption of the *Ofd1* gene in the mouse are thus more severe than those observed in humans from a clinical point of view. We hypothesize that this could be explained by the differential X‐inactivation pattern observed between the two species (Franco & Ballabio, 2006; Morleo & Franco, 2008). The escape from XCI in humans determines a biallelic expression with females displaying half a dose of the functional transcript in each cell. Next‐generation sequencing (NGS) technologies has demonstrated a significant degree of heterogeneity in the expression levels of genes escaping XCI, not only among individuals but also in different tissues from the same individual (Garieri et al., 2018; Tukiainen et al., 2017). In this scenario, OFD1 will escape XCI, at least partially, in some tissues and will undergo XCI at variable degrees in other tissues. This very probably influences the clinical expressivity of the phenotype in female patients. On the other hand, in the mouse where the gene is always subjected to XCI, females are mosaics and about 50% of cells will be lacking the expression of the gene. The severity of the phenotype in mice could be due to the requirement of at least one functional copy of the transcript in each cell (Franco & Ballabio, 2006; Morleo & Franco, 2008). Figure 3 depicts examples of the clinical differences observed between *Ofd1* mutant animals and OFD type I patients.

Interestingly, to add more complexity to the contribution of XCI to the clinical heterogeneity, at least two familial cases in which the mother and the daughter displayed contrasting XCI pattern have been described although it should be noted that the X‐inactivation studies were performed on DNA extracted from peripheral blood. In the first case, the mother presented a mild phenotype and a skewed X‐inactivation pattern whereas the daughter displayed a severe phenotype with random X‐inactivation (Thauvin‐Robinet et al., 2006). On the contrary, in a different familial case, the mildly symptomatic mother displayed a random inactivation while her daughter with the full OFD type I phenotype showed skewed XCI (Iijima et al., 2019).

There are no doubts that XCI plays a role in the phenotypic variability observed in this disorder. However, based on the recent data, XCI studies performed on peripheral blood samples do not necessarily reflect the XCI status in the affected tissues considering the variability observed (Garieri et al., 2018; Tukiainen et al., 2017). Hopefully, in the next few years, new tools will be available to ascertain the XCI status more appropriately in different tissues at a single cell level.

### 6.2 OFD1 mutations: Connecting phenotypes to genotypes

OFD type I is inherited as a dominant trait with male lethality thus only females are affected, while all the other conditions so far associated with *OFD1* mutations are inherited in a recessive fashion thus males are affected, and female carriers are asymptomatic. The OFD1 transcript comprises 24 exons and generates several splice variants among which the three main ones include the canonical transcript (NM_003611.2), and two additional RefSeq transcripts (NM_001330209.1 and NM_001330210.1). The OFD1 protein (UniProtKB 075665) has been extensively studied and different domains have been identified. A Lis1 homology (LisH) motif is found in the N‐terminal part of the protein. LisH motifs are commonly described in proteins, where they are believed to play a role in protein–protein interactions, dimerization, stability, and/or localization. Furthermore, this motif may be involved in the regulation of microtubule dynamics (de Conciliis et al., 1998; Ferrante et al., 2003; Gerlitz, Darhin, Giorgio, Franco, & Reiner, 2005). In addition, six putative coiled‐coil (CC) domains can be recognized in the central region of the protein where they contribute to homodimerization of the protein (Giorgio et al., 2007). More recently, analysis of the OFD1 protein sequence predicted a conserved LC3 interacting region (LIR) motif in a putative intrinsically disordered region at the C‐terminus of the protein. This LIR domain was then shown to be functional and to mediate OFD1‐dependent degradation of components of the unc‐51‐like kinase (ULK1) complex, a key player of the early steps of autophagosome biogenesis (Morleo et al., 2021).

The position of the domains and a schematic representation of the localization of the mutations found in OFD type I, PCD, JBS10, and RP23 patients are depicted in Figure 1.

As illustrated in Figure 1 and in Table 1, the mutations resulting in an OFD type I phenotype are mainly found within the first 17 exons of the transcript.

The mutations leading to PCD, instead, are clearly concentrated in the C‐terminal part of the protein (exons 20–21) although the patients described by Budny et al. (2006), presenting with signs of both OFD type I and PCD displayed a non‐sense mutation (c.2122‐2125dupAAGA; p.N711KfsX713) in exon 16. The nucleotide variations associated with JBS10 are localized to exons 17–21 although a few mutations in exons 3, 6, 7, 8, 9, and 11 have been reported. Most of the mutations are frameshifts, although also few missense, splicing, and inframe deletion mutations have been described (Table 1). The variants associated with RP23 patients include a missense mutation in exon 4 (c.358A>G; p.T120A), a deep intronic mutation causing the insertion of a cryptic exon spliced between exons 9 and 10 and resulting in a frameshift (IVS9+706A>G; p.N313fs.X330) and a missense variant in exon 22 (c.2953G>A; p.G985S).

All the information available to date suggest that haploinsufficiency may be the mechanism underlying OFD type I with a role played also by XCI levels to fully explain the variable clinical expressivity of the phenotype in female patients. The recessive inheritance of PCD, JBS10 and RP23 would be the result of loss of function changes impacting on the function of the pleiotropic OFD1 protein. How can we explain why mutations in the same exon (e.g., frameshifts in exon 20) or even exactly the same frameshift mutation (i.e., c.2789‐2793del TAAAAA; p.I930Kfs*8 in exon 21) cause either PCD or JBS10 which are two distinct ciliopathies with the first affecting motile cilia and the second immotile/sensory (primary) cilia? Moreover, what is the explanation for specific mutations (e.g., a frameshift mutation in exon 10) resulting only in a retinal phenotype only without extraocular manifestations?

Obviously, the situation is complex and several factors such as the presence of modifiers, the impact of tissue specific enhancers, the effect of activating or inactivating specific isoforms, and the extent of non‐sense mediated decay in different tissues may contribute. Several OFD1 interactors have already been identified and specific interactions, yet to be unveiled, may also contribute to the picture. Only the transcriptomic and proteomic study of the specific mutations and a thorough analysis at the genomic level may help to clarify the answers to these questions.

Another puzzling point is represented by the male lethality. If, as it seems, the hemizygous male cannot survive without OFD1, how do we explain the 14‐year old male twins with a missense mutation in exon 19 and renal cystic disease as the only clinical sign (H.‐W. Zhang et al., 2020)? What about the 9‐year old boy with intellectual disabilities, end stage renal failure (small kidneys no renal cysts), and retinal dystrophy in whom a 7.9‐kb deletion inherited from the asymptomatic mother and spanning exons 16, 17, and 19 was identified (Sharma et al., 2016)?

The majority of the mutations identified in living males are located from exon 17 onward (Figure 2). It is tempting to speculate that the first 16 exons of the transcript are necessary for males to survive. To answer this question, we should first be absolutely sure that all the variants identified in males up to exon 16 are indeed causative of the phenotype displayed by the patients. However, the validation of variants of unknown significance is one of the challenges of medical genetics in the NGS era and again detailed analysis of the single specific mutations will be necessary to confirm the pathogenicity of the variants.

## 7 CONCLUSIONS

Medical genetics is currently experiencing a very exciting and intense time, thanks to the opportunities provided by NGS technologies that will allow scientists to address complex biological questions, such as the ones presented in this article concerning the clinical manifestations derived from OFD1 impairment. This represents a paradigmatic example of the complexity of genetic diseases and of the different elements that can influence the clinical manifestations of inherited disorders. Unraveling the mechanisms underlying this complexity will contribute to explaining human complexity.

## CONFLICT OF INTEREST

None.

## Supporting information


**Appendix** **S1**: Supporting Information

## Data Availability

The data that support the findings of this study are available in Public databases containing mutation information such as ClinVar at https://www.ncbi.nlm.nih.gov/clinvar/.
