# Supplementary material for: OFD1 : One gene, several disorders
Source: Am J Med Genet C Semin Med Genet. 2022 Feb 2;190(1):57–71. doi: 10.1002/ajmg.c.31962 (PMC9303915; doi:10.1002/ajmg.c.31962)

Supplementary information

Schematic representation of the localization of the nucleotide changes identified in the OFD1 gene in OFD type I female patients. The program Protein paint (<https://proteinpaint.stjude.org/>) was used. Top panel, frameshifts mutations. Bottom, missense, non-sense, splicing and indel mutations are reported according to the color code. Th Protein and the RNA length are indicated at the top of each panel. The domains are shown and indicated following the nomenclature reported. Only the experimentally validated LIR in exon 21 is reported. Exons are represented as rectangles of different size in the bottom panel, number within exons (bottom panel) or along the protein length (top panel) indicate positions of amino acids. Exons, domains, RNA or protein lengths and mutations are in scale.


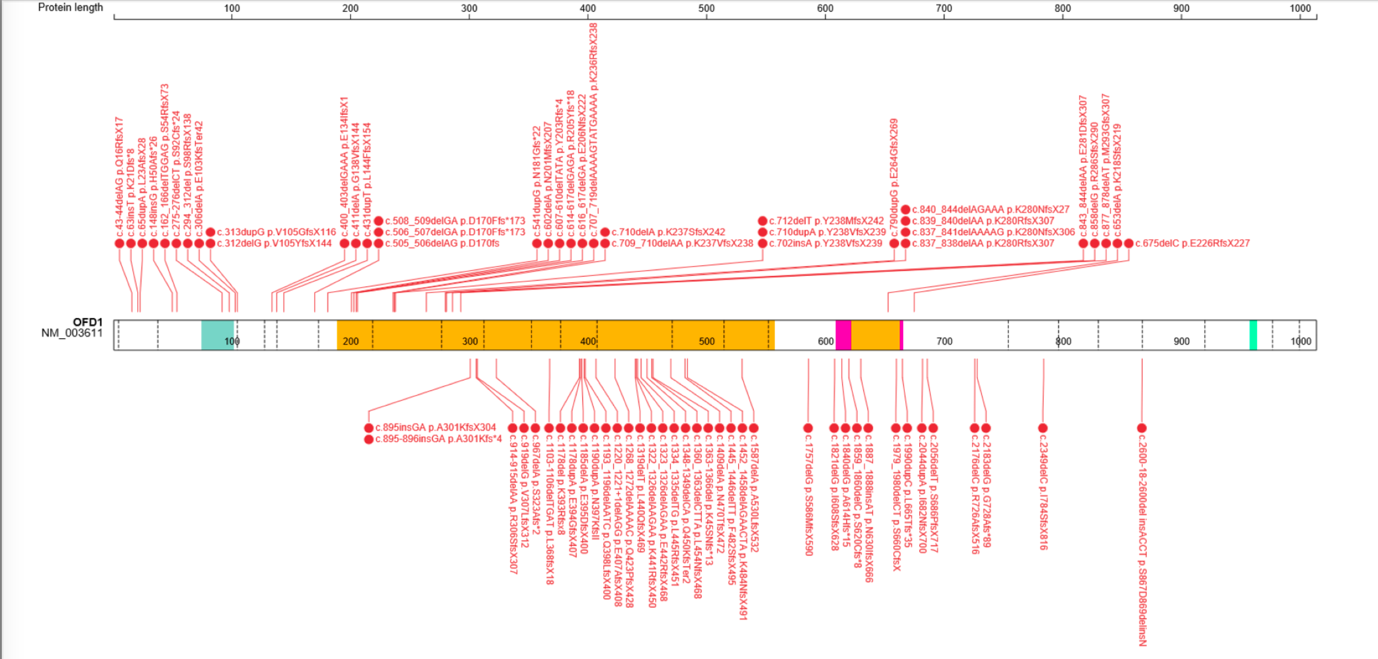


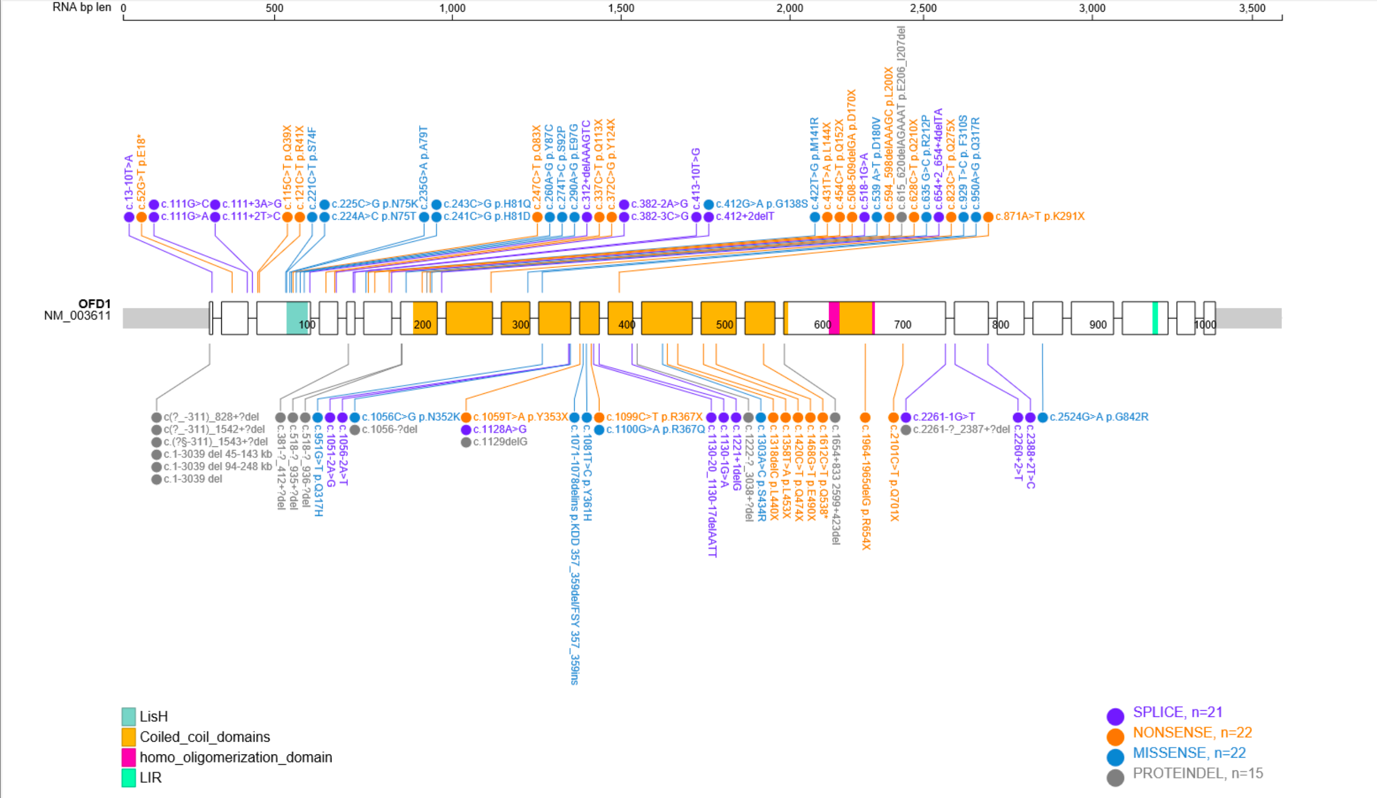

Supplement: Supplementary file 1 — Appendix S1: Supporting Information [file AJMG-190-57-s001.docx]
